# An imprint method to produce surface asperities for EHL and RCF experiments

**DOI:** 10.1016/j.mex.2020.101178

**Published:** 2020-12-08

**Authors:** Carl-Magnus Everitt, Bo Alfredsson, Martin Öberg

**Affiliations:** KTH Royal Institute of Technology, Solid Mechanics, Stockholm, Sweden

**Keywords:** Asperities, Contact mechanics, Elastohydrodynamic lubrication, Rough surface, Imprint

## Abstract

A method was developed for creating single well-defined surface asperities using an imprint technique. The proposed method enables:

• Creation of well-defined micrometre high asperities
• Creation of asperities which survived more than 35 million EHL contact cycles
• Damage tracing thanks to the possibility to control the damage initiation sites.

The technique is based on rolling a hard disc with indents against a soft disc for creating single surface asperities. The contact pressure causes plastic deformation forcing material into the indents to create the asperities. The height of the asperities can be controlled by adjusting the applied force. After initial reshaping during the run-in process, the asperities were strong enough to survive more than 35 million elastohydrodynamic lubrication cycles, which should be of great interest for the researchers who investigate rolling contact fatigue experimentally. The method could also aid the research on the run-in process by enabling tracing the development of specific surface defects. Since the method can produce high and strong asperities it might also prove useful for investigations how asperities deform under sever contact conditions.

## Graphical abstract

**Figure:**
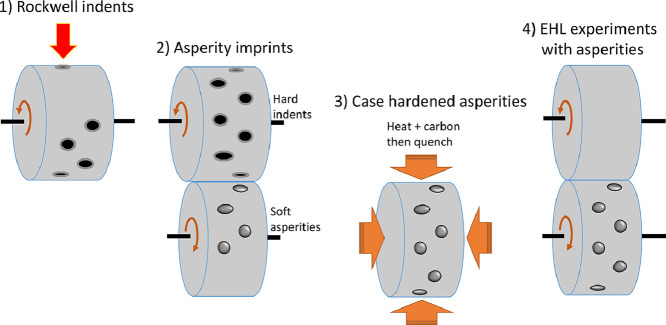

Speciications table

**Table:** 

Subject Area	Engineering
More specific subject area	*Rolling Contact Fatigue*
Method name	*Asperity Imprint Method*
Name and reference of original method	*NA*
Resource availability	*NA*

## Method introduction

A method was developed for manufacturing single asperities integrated into the hard contact surfaces of rolling contact discs for use in experimental investigations of the influence of single asperities on rolling contact fatigue (RCF) in hard lubricated and rolling contacts. The method is important for the experimental verification of the asperity point load mechanism for RCF [1], [2], [3], [4], [5] which suggests a plausible model for the initiation of surface initiated RCF.

A series of experimental RCF set-ups exist [6], ranging from product tests of bearings and gears, to surface experiments in twin-disc testers. Some experiments focus on the influence of dents [7] or grooves [8] on RCF and lubrication film properties. The reversed surface design, with individual ridges or asperities is harder to manufacture. Local material deposit is one method. It works for lubrication studies with limited cycle numbers and contact pressures. The challenge remains to deposit material that endure millions of over-rolling cycles at extreme RCF pressures.

Indentations have been used to create point defects by numerous researchers. For instance Ville and Nelias [9], Coulin et al. [10], Lorösch [11] and Dommarco et al. [12] have investigated effects of indents on RCF. The experiments show that indents reduce the fatigue lives of the surfaces by creating pressure peaks. The indents are often connected with a circumventing pile-up ring. Following the asperity point load mechanism, RCF cracks initiate at the trailing edge of the pile-up and grow in the forward rolling direction at a shallow angle. However, these undulation become 5 to 100 times larger than asperities in applications; it contains a deep dent which serves as an oil reservoir; the material structure is heavily deformed, which affects fatigue properties and residual stresses; it can be hard to separate the effects of the pile-up from those of the indent on RCF life. Thus, direct hardness indents are not well suited for damage studies in applications.

The literature describes methods to adjust the surface properties for elastohydrodynamic lubricated (EHL) experiments. Chromium sputtering techniques have been employed to create single asperities for experiments on EHL effects. Kaneta and Cameron [13], and Choo et al. [14] studied three-dimensional chromium asperities, also called bumps, in EHL contacts. The asperity height was however limited to maximum 260 nm in these investigations. Sputtered asperities also have the challenge to remain during testing at heavy RCF conditions.

Line defects have been investigated by for instance Venner et al. [15] who studied the amplitude attenuation of sputtered waviness in an elliptical contact. Höln et al. [16] produced a wavy surface pattern by grinding and etching. Much focus has also been directed towards the deformation of transverse ridges. Guangteng et al. [17] created a 150 nm high chromium ridge on a steel ball by sputter-deposition. Sperka et al. [18] used a sputter-etching process on a steel ball to create 200 nm high ridges. From a fatigue point of view, essential differences exist between line and point defects with respect to contact mechanics and stresses. Since surface initiated RCF typically initiates at single points, it is essential to understand the local phenomena at the contact of point defects.

Thus, the presently proposed method to produce single surface asperities fills a gap in the methodology to design and modify hard contact surfaces. The main focus was on RCF where the asperities can be used in experimental studies on fatigue initiation and the subsequent damage evolution. The method to implement local surface modifications could however be of value for other research areas. The point contact has implications on the local stresses and the detailed position of generated heat. The method may assist in characterizing the run-in process of surface asperities, especially for cases when the surface profile remains but the metal contact is removed [19]. The shape changes of surface defects during run-in can be studied and the asperity attenuation theories [20] for surface roughness can be investigated.

The method could also useful for EHL experiments on the lubricant flow around single well-defined asperities and investigations on the separation of the real and complementary effects caused by point asperities [21]. The method enables experimental verification of numerical results that show that it is the trailing edge of the asperity that breaks through the lubricant rather than the asperity peak [22].

The proposed imprint method was here used to create asperities with height ranging from 4 to 15 µm but the method can be used to manufacture both lower and higher asperities. The asperity diameter was varied between 100 and 200 µm, but a wider range is possible. The asperities were fully integrated in the surface with the same material as the substrate and without any internal interfaces. Hence, the present asperities have the potential to remain during heavy RCF testing. The method allows numerical simulated contacts with asperities to be compared to experiments, which is highly south after by researchers investigating RCF. If it is used for studies on EHL, then lower asperity hights than the ones manufactured here would be preferred, which would be attained by lowering the imprint contact force.

## Procedure

Three disc geometries were needed for the asperity imprint method and subsequent contact experiments. The first disc is called the indent-disc since it had the indents. The second disc geometry had the asperities and is thus named asperity-disc. Thirdly, the EHL experiments required a counter-disc. Manufacturing and experiments included:

(1) Manufacturing blank discs.
(2) Harden the indent-disc.
(3) Create Rockwell cone indents on the indent-disc.
(4) Remove pile-up around the indents.
(5) Imprint the asperities by rolling the soft blank disc against the hard indent-disc.
(6) Harden the asperity- and counter-discs.
(7) Perform EHL experiments with the hard asperity- and counter-discs.

**Fig. 1:**
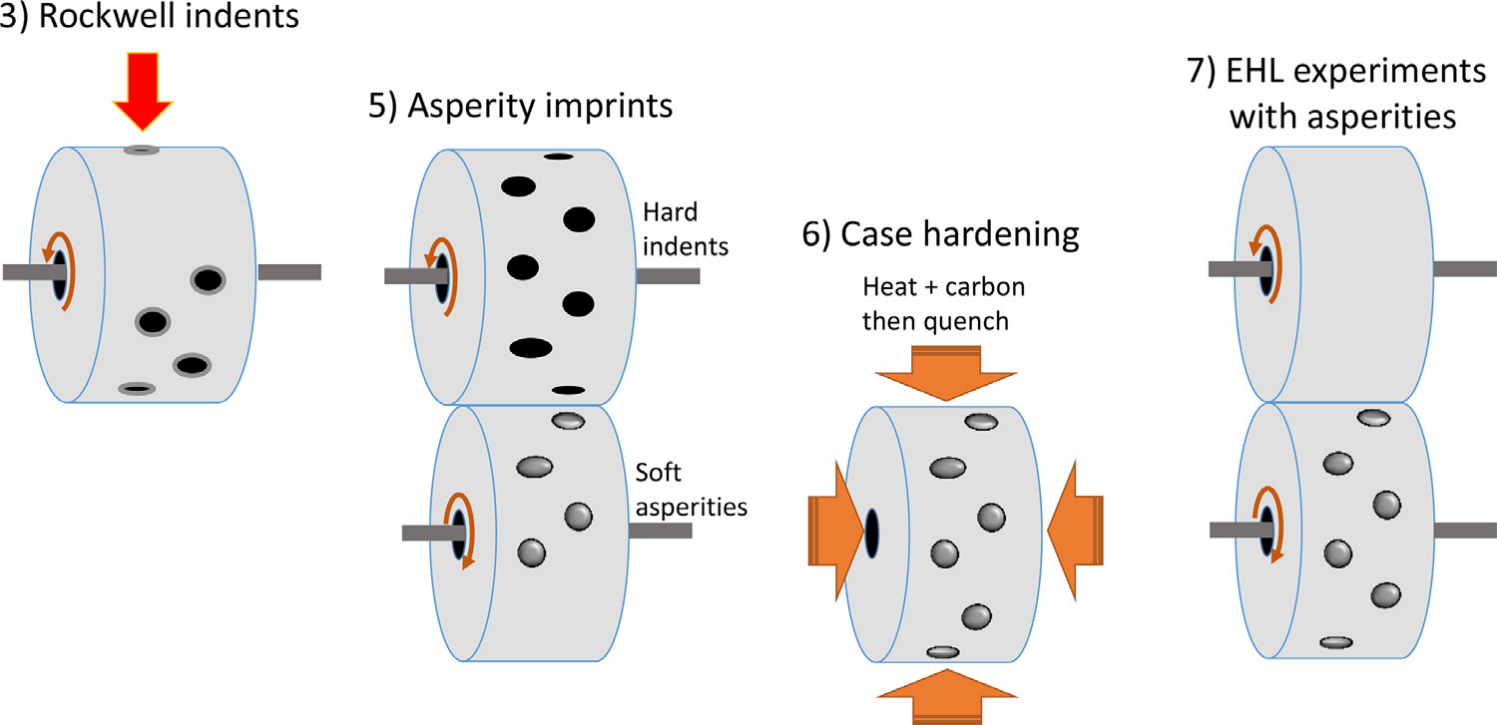
The key steps of the imprint method for creating single well-defined asperities.

Steps (3) and (5) to (7) are visualized in Fig. 1.

### (1) Manufacturing blank discs

Three types of disc blanks were manufactured in two different materials. The indent-discs were made of an easily hardened tool steel following AISI 01 or DIN 1.2510. The exact quality can be varied relatively freely. The asperity- and counter-discs were made of a gear steel that follows the Swedish standard SS142506 (ISO 20NiCrMoS2). The indent- and asperity-discs were without transverse crowning for flat well controlled imprint operation without remaining imprint groove. The indent-discs should be slightly wider than the asperity-discs to ensure that the asperity-disc does not roll over the edge of the indent-disc during the imprint operation. Such events will cause unwanted line indents from the indent-disc on the asperity-disc. The counter-disc was designed with a transverse crown to remove edge effects and control the contact pressure during the subsequent RCF experiments. Drawings of the discs are presented in Appendix A. Appendix B contains material data and the stress-strain curve for SS142506. The material data was used in FEM-simulations to predict the asperity heights.

### (2) Heat treatment of indent-disc

The blank indent discs were heated in air to 800 °C for 30 min and then quenched in oil at room temperature. This yielded the hardness 56 ± 5 HRC. The asperity-discs could not be hardened at this stage as they had to be soft when the asperities were imprinted using plastic deformation.

### (3) Make indents

A Rockwell cone was used for circular indents and the subsequent imprint of asperities. The indentation load was 150 N. It created indents with diameter *D* ≈ 250 µm in the hardened indent-disc.

**Fig. 2:**
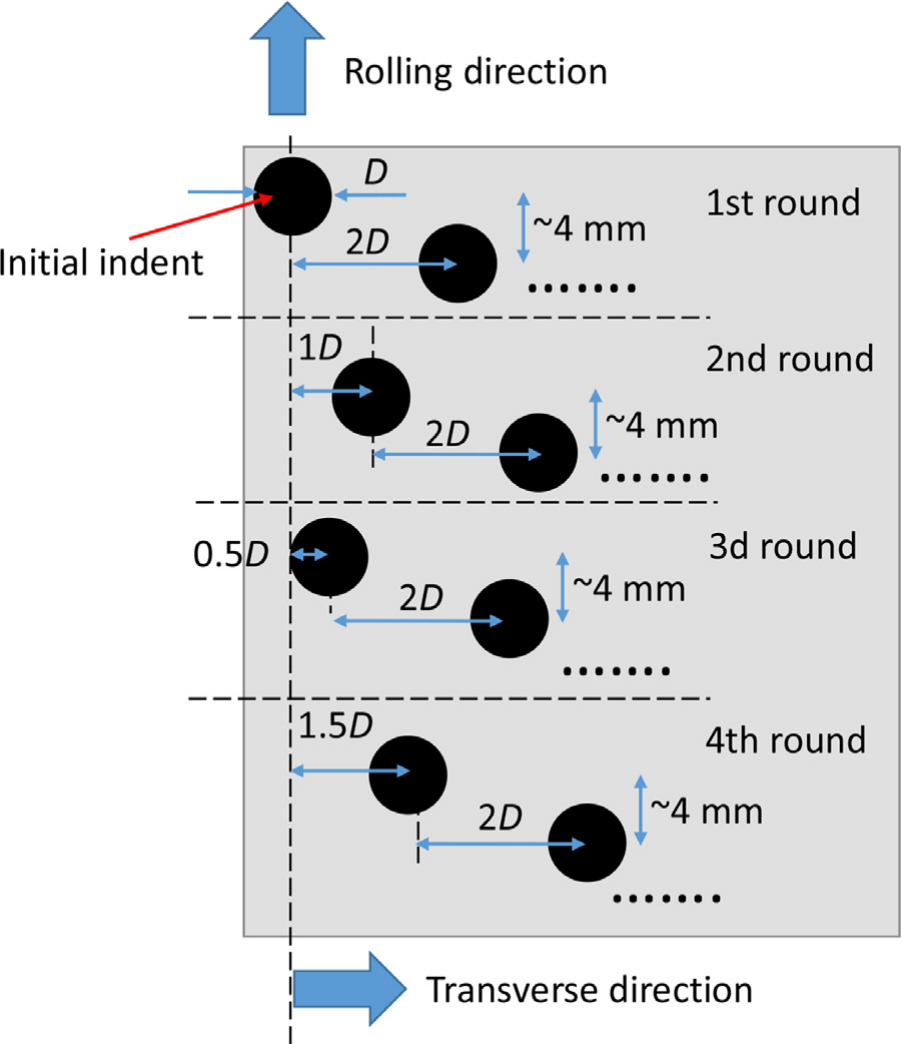
Indent-disc with the indent positioning method.

**Fig. 3:**
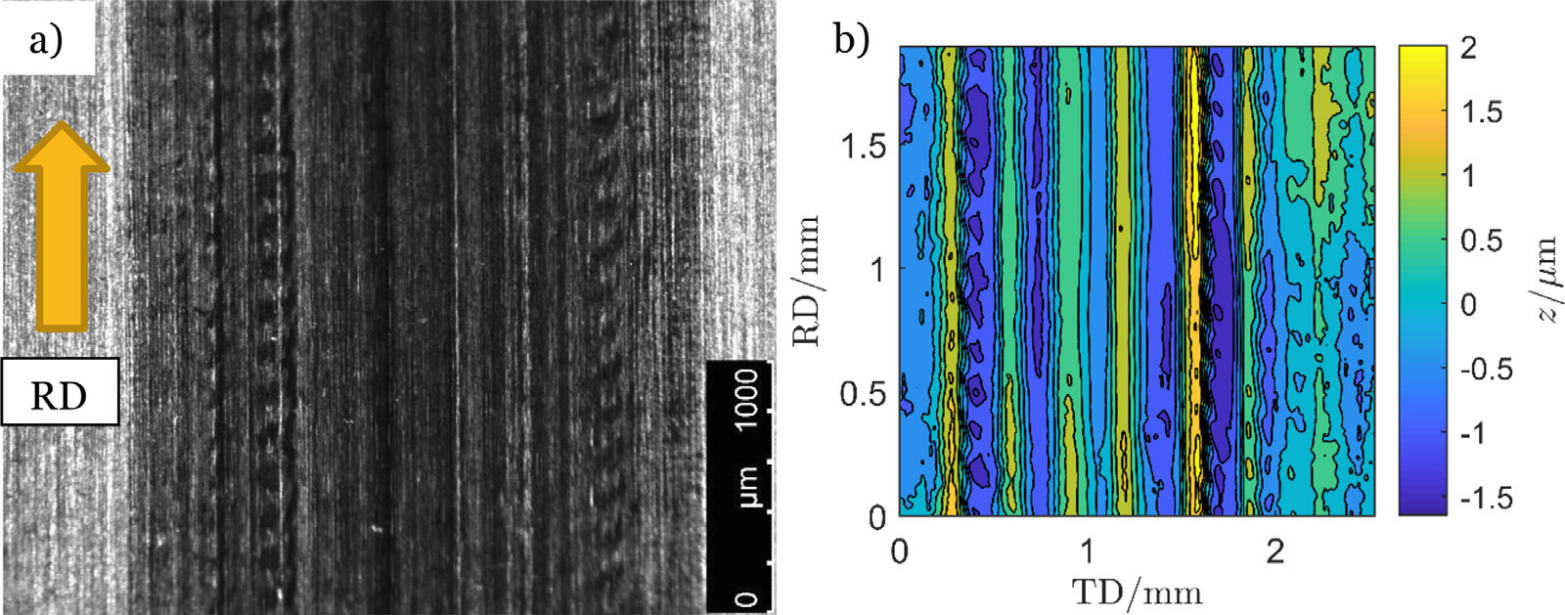
Tracks caused in the counter-disc by 15 µm high randomly placed asperities.

The indents were carefully placed in the axial, also denoted transverse, direction of the discs. The positioning method in Fig. 2 is proposed in order to obtain even wear of the counter-disc and even loading of the asperities in the subsequent EHL or RCF experiments. If the indents and subsequent asperities are randomly placed, then they may create tracks and ridges in the counter-disc during the rolling contact or EHL experiment. Fig. 3a shows a picture and Fig. 3b a contour plot of the surface structure of a counter-disc after an experiment where the asperities on the asperity-disc were 15 µm high and randomly placed. The maximum Hertzian pressure was 2.3 GPa and the experiment was performed for 11 million load cycles. The asperities caused almost 3 µm deep groves in the counter-disc surface. A fourth order polynomial was used to remove the surface curvature in both the rolling direction (RD) and the transverse direction (TD) for the contour plot in Fig. 3b.

The asperity positioning method in Fig. 2 consists of four steps. In the first step an initial indent was created at one side of the disc surface. Thereafter, the indent position was shifted in the TD two times the indent diameter, *D*, for each new indent until the other side of the indent disc was reached. At each transverse shift, the position for the next indent was also moved 4 mm in the RD. The distance between the asperities in the RD could be chosen relatively free but should ensure that the EHL contacts and the stresses around the asperities do not interact with each other. Note that this distance was reduced for visualization purposes in Fig. 2. A second round of indents was then created with the initial axial position one indent diameter from the initial indent of the first round. Thereafter, a third round was added with the initial axial offset set to a half indent diameter. The pattern was completed with a fourth round of indents. It started one and a half indent diameter from the axial position of the first initial indent. The first indent should be placed so the asperity it forms ends up just outside the EHL contact in the experimental investigation. The positioning method should extend transversely so far that the last indent forms an asperity outside the other side of the rolling contact.

**Fig. 4:**
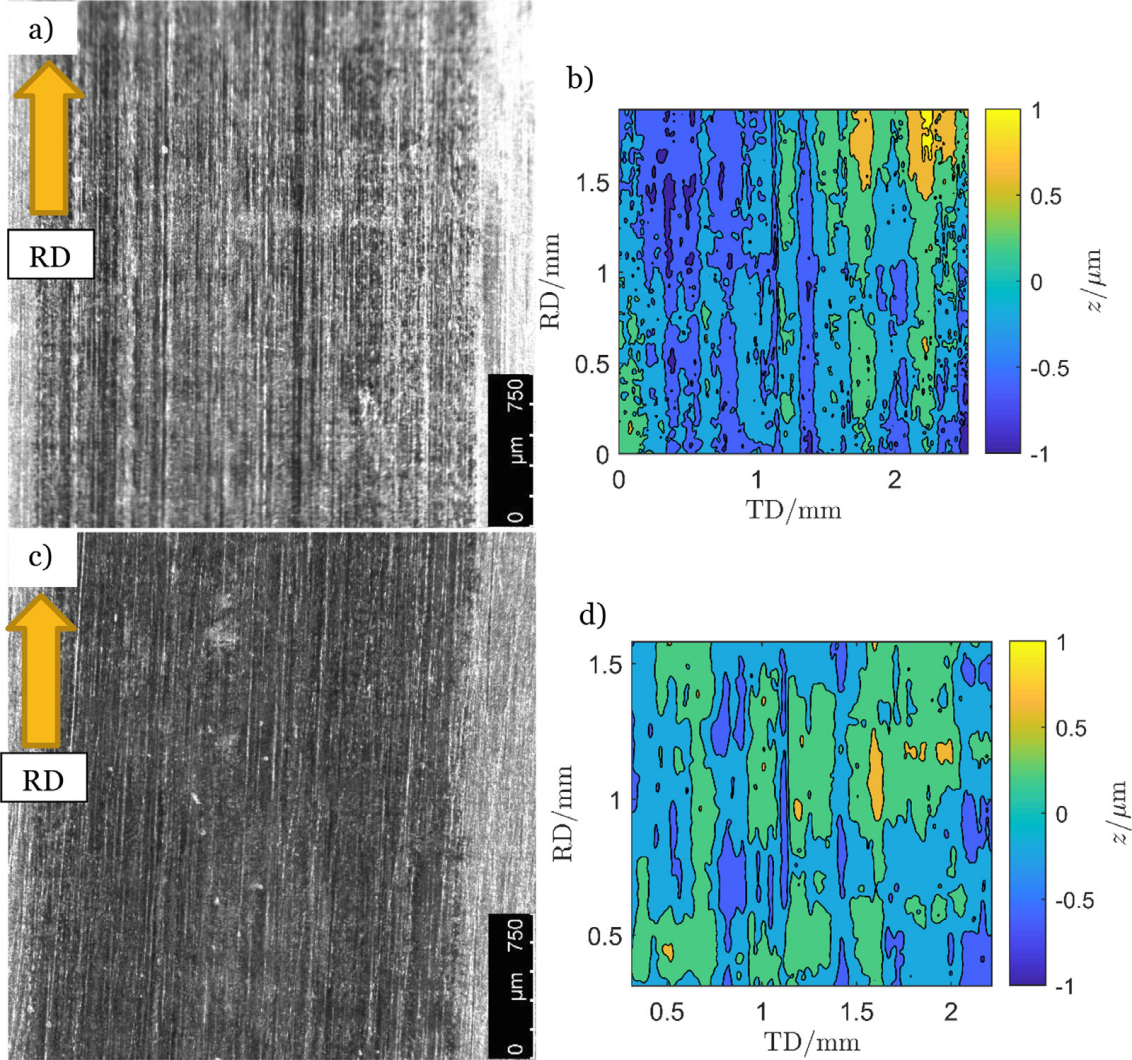
Surface measurement of the counter-disc after experiment termination. The axial distance between the asperities in a) and b) were random and in c) and d) they were arranged in the pattern in Fig. 2.

Fig. 4 shows two counter-discs which were used to apply the contact load to asperity-discs with 4 to 5 µm high asperities. The results in Fig. 4a and 4b were produced by randomly placed asperities while those in Fig. 4c and 4d were the outcome from experiments where the asperities were placed using the structured method outlined in Fig. 2. All other conditions were identical between the experiments. In this case the carful positioning of the asperities reduced the profile height, from highest peak to deepest valley, of the tracks in the counter-discs with 50%, from 2 to 1 µm.

### (4) Remove pile-up

Depending on the strain hardening of the material, the rim of the indent may follow the indent, a so called sink-in, or the rim may rise, creating a protruding ring that circumvents the indent, a so called pile-up. If the indent results in a pile-up, then this will be imprinted as well as the indent into the asperity-disc surface.

**Fig. 5:**
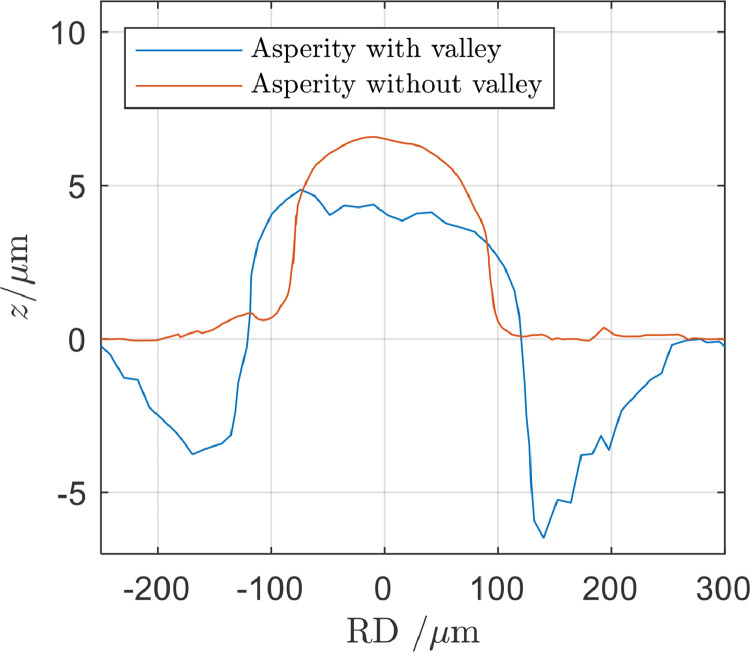
Cross-section of two asperities. One where the pile-up around the imprint indent had been removed and one where it had not been removed, which resulted in a valley that circumvented the asperity.

The indent-discs were therefore gently ground in order to remove any pile-up around the indents. One set of asperities were created before the pile-up was removed to visualize the difference, see Fig. 5. The pile-up in this experiment caused a valley with a depth of around 5 µm while no valleys were found if the pile-up was removed before the asperities were created.

### (5) Asperity imprint

**Fig. 6:**
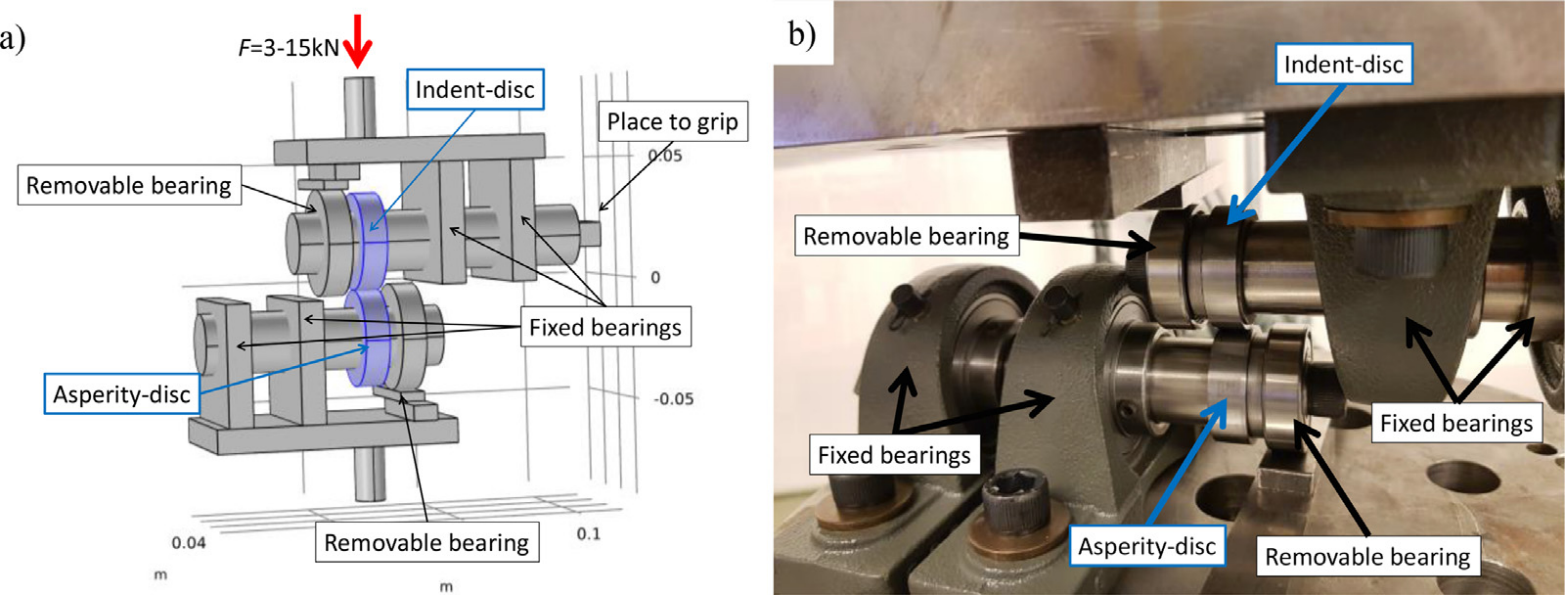
(a) Set-up of fixtures used for creating asperities. The asperity- and indent-discs are marked in blue. (b) Actual set-up.

A fixture was designed and manufactured for the imprint of the asperities. The fixture was placed inside a servo-hydraulic test machine which allowed force-controlled loading on the specimens. The hardened indent-disc was pressed against the asperity-disc to the selected imprint force and then manually turned one full round using a lever attached to the grip in Fig. 6a. Thanks to the force control option of the test machine the imprint pressure remained constant throughout the revolution. The set-up is drawn in Fig. 6a and a close-up picture is presented in Fig. 6b. One removable bearing was added outside each of the discs to create additional support that minimized the radial deformation of the shafts, see Appendix C for drawings of the shafts. Without the extra support the discs moved slightly in the transverse direction during imprint. To further reduce the radial displacement the distances between the bearings and the discs were minimized. By using low friction bearings, the asperity-disc followed the intent-disc due to dry friction in the imprint contact. It is also important that the shafts are parallel to each other. This was achieved by manually adjusting one of the fixtures after both discs were mounted but before the imprint force was applied. The alignment of the shafts was then verified by visual inspection.

**Fig. 7:**
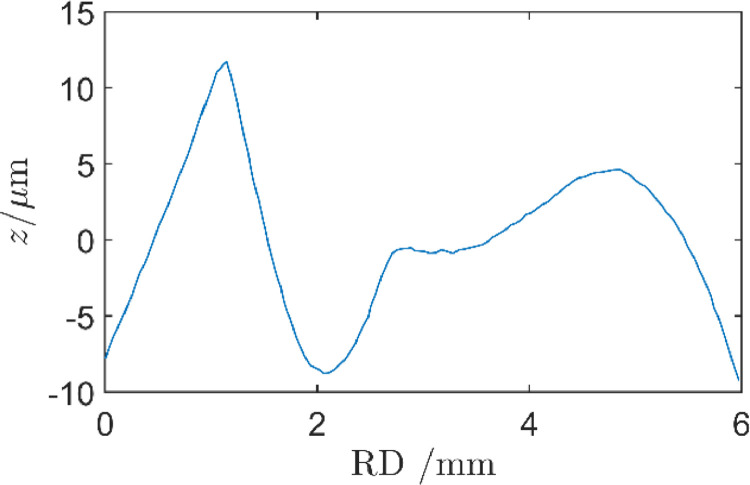
Longitudinal surface profile of asperity-disc at the start and end point of the imprint contact. The curvature of the disc was removed.

The load was applied after the discs were mounted and the shafts were aligned. The indent-disc was then turned one round during which the asperity-disc followed due to dry friction in the contact. Finally, the load was released, and the asperity-disc was removed for inspection. A slight line pile-up was produced in the asperity-disc surface at the point of first contact by the indent-disc since the load was applied when the discs were standing still. The line pile-up around the location of initial and last contact coincided since the disc was turned one full revolution. Fig. 7 presents a longitudinal profile across the initial contact pile-up for a disc that had been subjected to an imprint force *F* = 10 kN. The presence of such line defect could, if too large, introduce vibrations during the subsequent RCF experiment, which in turn may affect RCF life as illustrated by Li and Kolivand [23]. The present defect had half the hight and 25 times the longitudinal extension in the rolling direction compared to the smallest defect studied by Li and Kolivand. Since the smallest defect was barely noticeable in the reference [23] and the present line defect was 50 times smoother, it was judged as acceptable. The resulting vibrations in the twin-disc machine used in step 6 was monitored and found to be within the allowable range for experiment. Since it was positioned away from the asperities it did not affect the present RCF experiments.

**Fig. 8:**
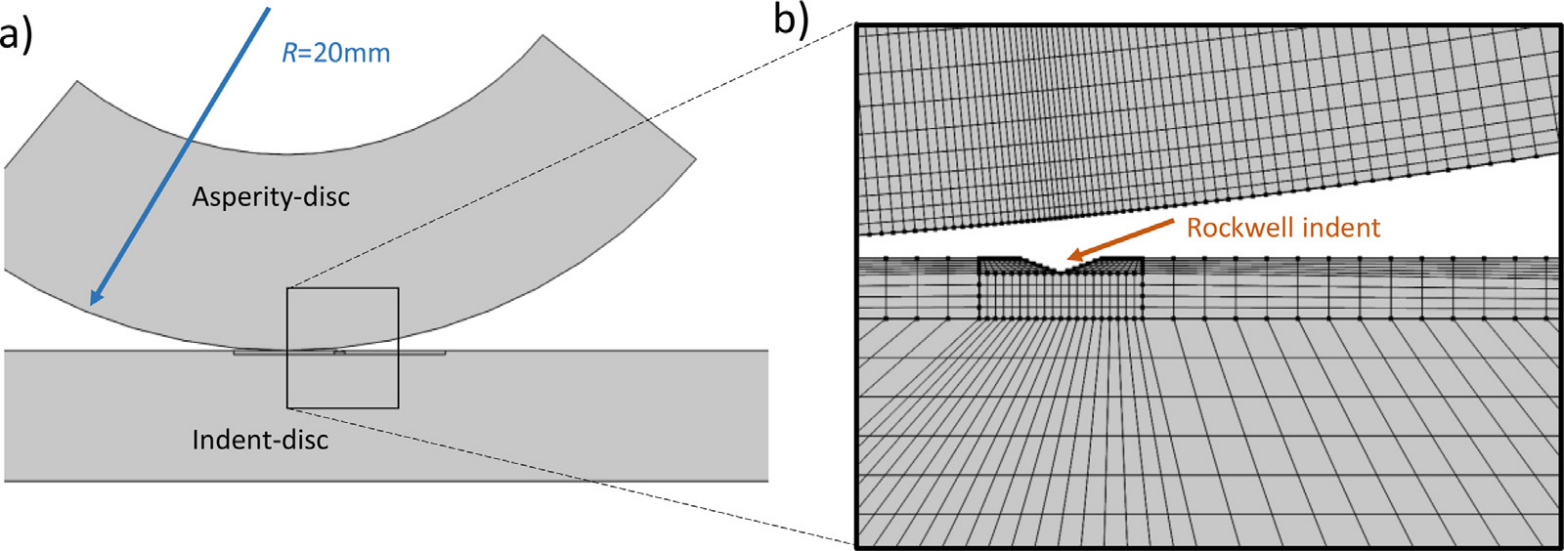
FEM geometry and mesh: (a) full geometry, (b) detail of the mesh at the Rockwell indent.

**Fig. 9:**
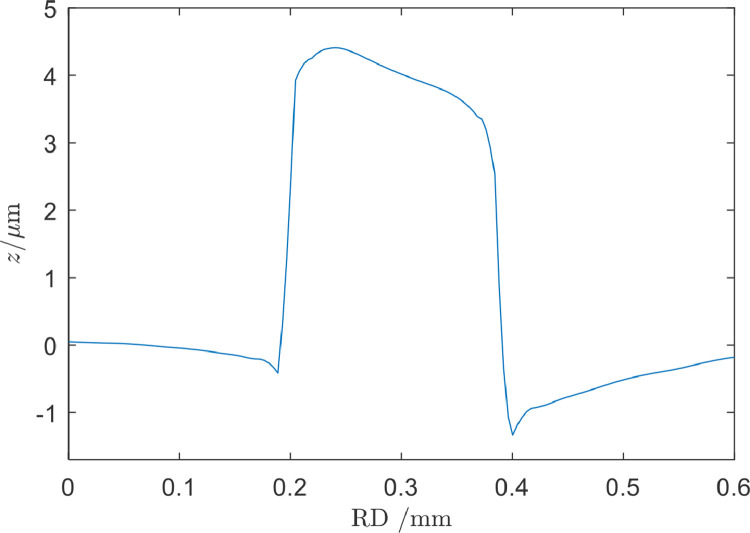
FEM prediction of the asperity shape.

To estimate the imprint force *F* a two-dimensional plane strain FE-model was used with material parameters from Appendix B. Some predictions of the relation between applied force *F* and the asperity heights *δ* were drawn from the simulations. The commercial software Comsol Multiphysics [24] was used for the simulations. To simplify the geometry the indent-disc was represented with a flat surface while the radius of the asperity-disc was 20 mm, which kept the effective Hertz contact radius. Fig. 8 presents the geometry and mesh. The loads were applied as prescribed displacements. First the asperity-disc was pushed down 25 µm which corresponded to an imprint force of *F* = 6 kN in the experiments. Then the asperity-disc was rolled over the indent and finally it was lifted to release the contact. The resulting asperity shape is presented in Fig. 9. Note that the left side of the asperity is slightly higher than the right. This was also found when measuring some, but not all, produced asperities, see Fig. 5. The size of the predicted asperity in Fig. 9 is comparable to the experimental results in Table 1.

**Table 1:** Asperity heights *δ* from different loads *F* and indent widths *ω*.

*F/*kN	*ω*/µm	*δ*/µm
10	200	15
8	200	8
4	200	5
7	220	8
15	100	7
10	100	5

The relations between asperity height *δ*, imprint load *F* and indent widths *ω* are presented in Table 1 for the present materials. After manufacturing the asperities, the surface roughness *Ra*, excluding the asperities, was measured to 0.1–0.2 µm for the asperity-discs, independent of the applied imprint force. Since the indent-disc was hard and the asperity-disc was soft during the imprint operation, the indent-disc could be used for asperity printing on several asperity-discs, which is useful for investigations where the same asperity pattern is desired.

The size of the present asperities ranged from *δ* = 5 − 15 µm with about *ω* = 200 µm. A reduced indentation load would result in a smaller indentation, which would primarily reduce the asperity width after the imprint operation. Reduced imprint load *F* would primarily decrease the asperity height *δ*. Thus, the lower limit for asperity size will be set by approaching the surface roughness. Larger asperities could in theory be attained by increasing indentation and imprint forces. The limitations will be given by fixture deformation and size of the line defect at imprint start. The literature [23] may give guidance on the limitation of the line defect with regards to vibrations. For RCF experiments in twin-disc machines the experiments showed that the largest asperity with *δ* = 15 µm was in practice too high.

### (6) Heat treatment of asperity and counter-disc

The asperity- and counter-discs were both case carburized at 930 °C with 1.1% carbon atmosphere for 5 h and then equalized at 850 °C with 0.75% carbon for 1.5 h. Thereafter, the specimens were quenched in an oil-bath for 30 min. The specimens were then drained of any oil for 15 min and rinsed for 30 min at 70 °C. Finally, the specimens were tempered at 170 °C for 2 h.

The hardening procedure resulted in the surface hardness 61 HRC and a case depth of 1.24 mm. At these conditions, the asperity shape was intact but the surface roughness outside the asperities increased to *Ra* ≈ 0.4 µm. Since the asperities were quite small compared to the roller no heat treatment deformation developed relative to the roller. The heat treatment did however slightly change the shape of the interior disc holes. The holes were therefore adjusted with a wire electrical discharge operation to their original shapes.

### (7) EHL experiments

The asperity-discs where run against the counter-discs in a Wazau UTM 2000 twin-disc machine for up to 35 Mcycles. The experiments were performed with either a non-commercially available additive free (neat) polyalpha-olephin, PAO, oil provided by Agrol Lubricants in Sweden or a non-commercially available low additive mineral oil called Turbo TT9 provided by SKF in The Netherlands. The lubricant properties are presented in Table 2. Data for the experiments are presented in Table 3.

**Table 2:** Lubricant parameters for the oils.

Oil	Parameter	Symbol	Γ = 20 °C	Γ = 40 °C	Γ = 100 °C
Turbo TT9	Kinematic viscosity	*η*_k/_mm^2^/s	–	9	2.3
Turbo TT9	Density	*δ/*kg/m^3^	870	–	–
Turbo TT9	Barus exponent	*α/*GPa	30	–	22
PAO	Kinematic viscosity	*η*_k/_m^2^/s	–	104	15.4
PAO	Density	*δ/*kg/m^3^	836	–	–

**Table 3:** Data for the experimental set-up.

Parameter	Symbol	Set-up 1	Set-up 2
Max Hertzian pressure [25]	p_H_/GPa	2.3	2.3
Contact half width in RD [25]	*a/*mm	0.33	0.33
Contact half width in TD [25]	*b/*mm	1.2	1.2
Surface speed of asperity-disc	*v*_1/_m/s	5.7	5.9
Surface speed of counter-disc	*v*_1/_m/s	6.3	6.3
Lubricant bulk temperature	*Γ/*°C	90	90
Initial surface roughness (excluding asperities)	*R_a/_*µm	0.4	0.4
Lubricant		PAO	Turbo T9
Minimum film thickness [26]	*h*_min/_µm	0.4	0.1
Lambda-ratio (excluding asperities)	*λ/*-	1	0.25
Initial height of asperities	*δ/*µm	5–15	5
Initial width of asperities	*ω/*µm	180	180
Asperity pattern		Random	Structured
Material parameter [27]	*G/*10^3^	3.5	3.5
Speed parameter [27]	*U/*10^−12^	66	8.0
Load parameter [27]	*W/*10^−5^	8.8	8.8
Hardness of test discs	HRC	61	63
Hardness depth of test discs	*d*_H/_mm	1.2	2.2

#### The asperity-discs

**Fig. 10:**
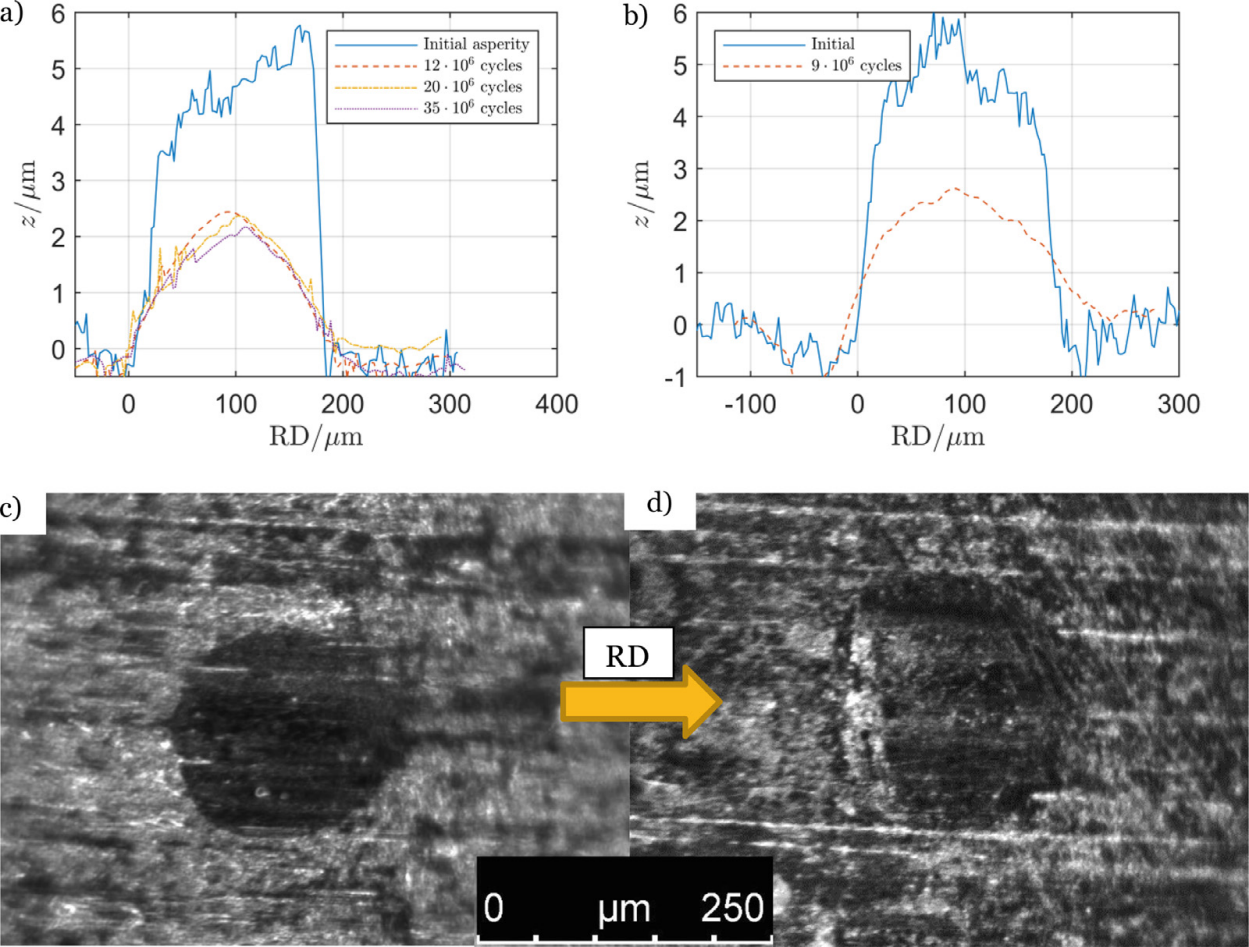
Development of asperities with original height *δ* = 5 µm and experimental set-up in Table 3: (a) randomly distributed and (b) evenly distributed according Fig. 2. Pictures of an asperity in each disc in (c) and (d).

One goal of the initial twin-disc rolling contact experiments was to test the endurance of the produced asperities. The experiments used the two set-ups in Table 3. The experiment presented in Fig. 10a and Fig. 10c followed set-up 1 with 5 µm high asperities while the experiment presented in Fig. 10b and Fig. 10d was performed according to set-up 2, also with *δ* = 5 µm. For both set-ups the final asperity height was about 2 µm at the end of the experiments. The counter-disc surfaces of these experiments are presented in Fig. 4. The counter-disc from the experiment in Fig. 10a is presented in Fig. 4a and the counter-disc from Fig. 10b is exhibited in Fig. 4c. Pictures of the asperities and the nearby surface were taken through a light optical microscope (LOM), Leica M205 FA, and camera, Hamamatsu C11440. An asperity from each experiment is presented in Figs 10c and 10d. The asperities are the dark round shapes in the middle of the pictures.

The maximum Hertz contact pressure p_H_ = 2.3 GPa used in the experiments corresponded to a high contact pressure in gear applications. The experiment illustrated in Fig. 10a was interrupted at some points to measure the development of the asperity profile. The first interruption was after 12 Mcycles, at which point the final asperity height and profile had been achieved. Comparing Fig. 10a and 10c with 10b and 10d no noticeable difference was detected in the height or profile except that some micro-pits had developed on the leading edge of some asperities in the experiment following set-up 2. One of these micro-pits is presented in Fig. 10d. The asperity in Figs 10a and 10c had survived 35 Mcycles and the one in Figs 10b and 10d had survived 10 Mcycles without severe pitting. If RCF is the research goal, then one way to increase the pitting risk would be to rise the slip, which was relatively modest, see Table 3.

**Fig. 11:**
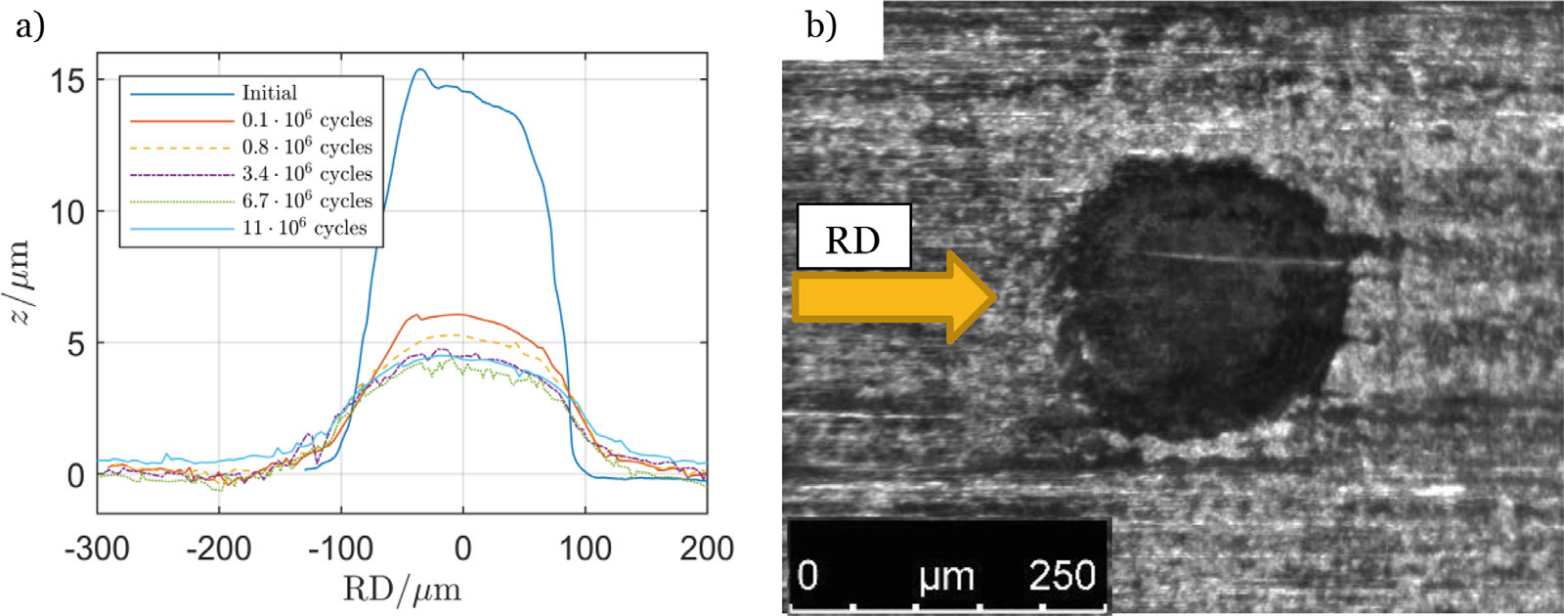
Experiment with *δ* = 15 µm: (a) profile evolution with cycle numbers along centre-line and (b) an asperity after 11•10^6^ cycles.

The imprint method was also used to manufacture 15 µm high asperities, such as in Fig. 11a. These were tested at the same conditions as the experiment in Fig. 10a. The slightly higher left side of the initial asperity was due to the rolling direction of the imprint procedure. The asymmetric shape with the higher left side agreed with the simulated shape in Fig. 9.

Fig. 11a includes the profile evolution of one asperity. The first interruption of the experiment was after 0.1 Mcycles for the profile measurement. The figure illustrates that almost all shape changing deformation occurred during run-in when the height was reduced from 15 to 6 µm. After run-in the shape and hight remained relatively steady.

Some micro-pits developed at the leading edge of theses asperities. They were similar to the one presented in Fig. 10d but in some cases they grew to be almost as large as the asperities. Fig. 3 presents the counter-disc surface with asperity tracks from this experiment. The distance from peak to bottom of the tracks was measured to 3 µm.

#### The counter-discs

**Fig. 12:**
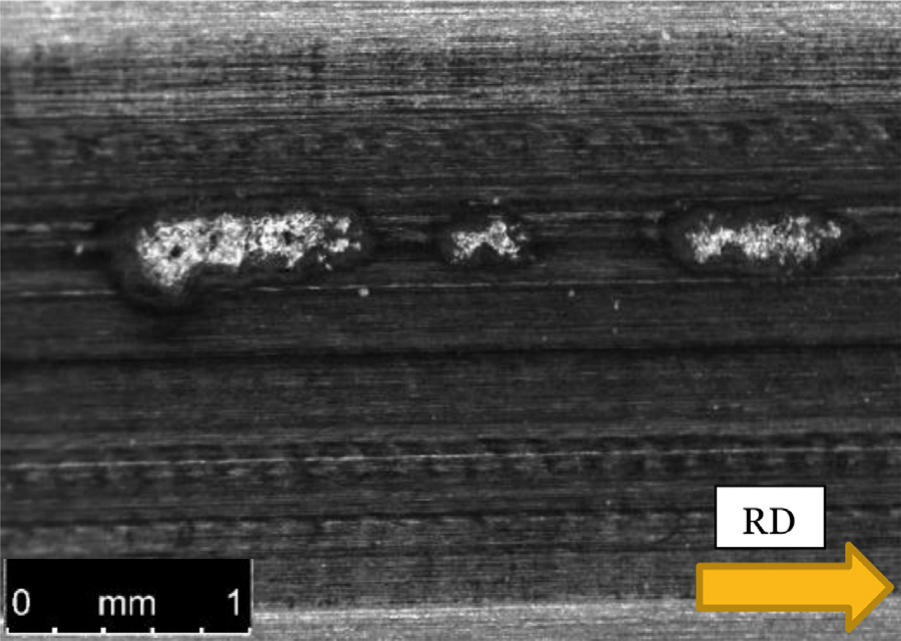
Pits in the counter-disc caused by high asperities, *δ* = 15 µm, and high contact pressure, *p*_Hertz_ = 2.3 GPa.

The asperities affected the surface of the counter-discs which in turn might affect the experimental results. Fig. 3 and Fig. 4 show asperity tracks on the counter-discs, which in Fig. 3 was 3 µm deep. In the experiment with 15 µm high asperities, three distinct micro-pits developed, see Fig. 12. Surface measurements revealed that the pits were about 20 µm deep. The impact on the counter-disc depended on the asperity size and shape as well as the asperity pattern (structured or random). It should be noted that these pits in the counter-disc were an exception, but they illustrate what may happen at too severe conditions, such as too high asperities and contact pressures.

## Conclusions

The imprint method could create single well-defined asperities in the micrometre range by rolling a hard disc with prefabricated indents against an unhardened disc in a ductile material. The sub-sequent case-carburizing heat-treatment of the asperity-disc merged the asperity material with the disc surface. After the heat-treatment, the asperities became fatigue resistant and could survive more than 35 million load cycles at high contact pressures.

The asperities underwent large shape changes during run-in. The initial asperity height decreased with about 50%. The part of the asperities that survived run-in was however able to withstand at least 35 million load cycles. The strategy for positioning asperities in the axial direction was found to be important. Careful consideration of the axial asperity positions clearly decreased the depth of wear tracks in the counter-disc.

The critical sites for damage were known from the asperity manufacturing. Therefore, the damage development at critical sites could be traced through the experiments.

The Rockwell C indents into the hardened indent-disc could, depending on the strain-hardening of the material, result in so called pile-up around the indent with material protruding from the surface. Careful grinding of the surface with indents before the imprint operation removed the pile-up and the valley that otherwise would circumvent the asperity.

## Declaration of Competing Interest

The authors declare that they have no known competing financial interests or personal relationships that could have appeared to influence the work reported in this paper.

## Figures and Tables

**Fig. A1:**
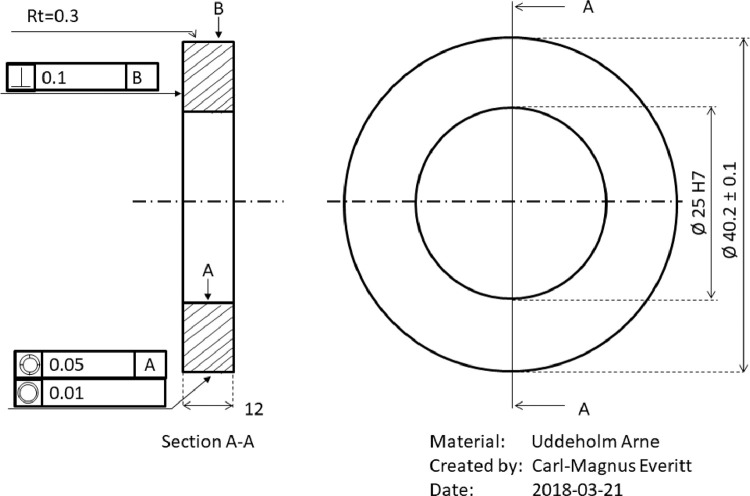
The indent-disc.

**Fig. A2:**
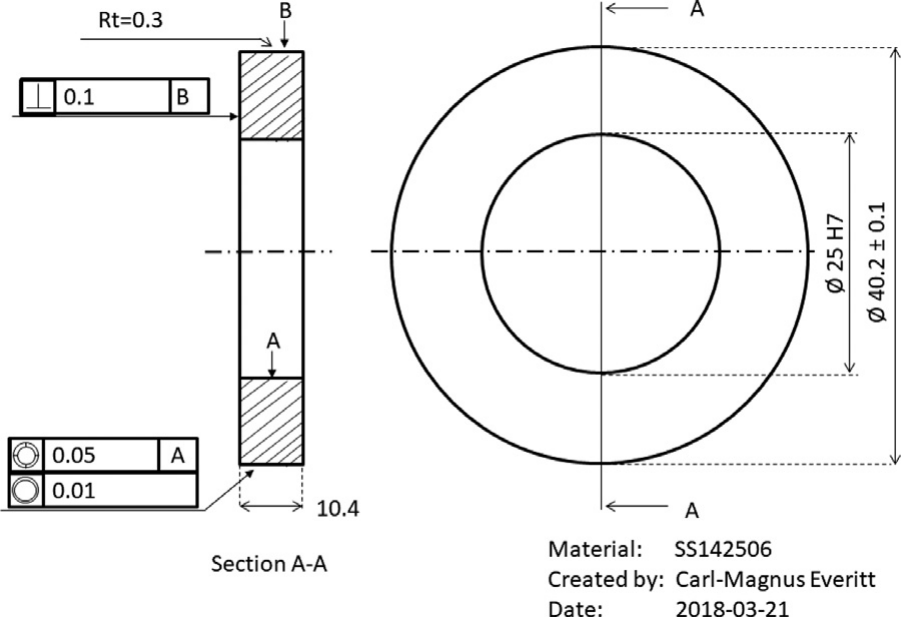
The asperity-disc.

**Fig. A3:**
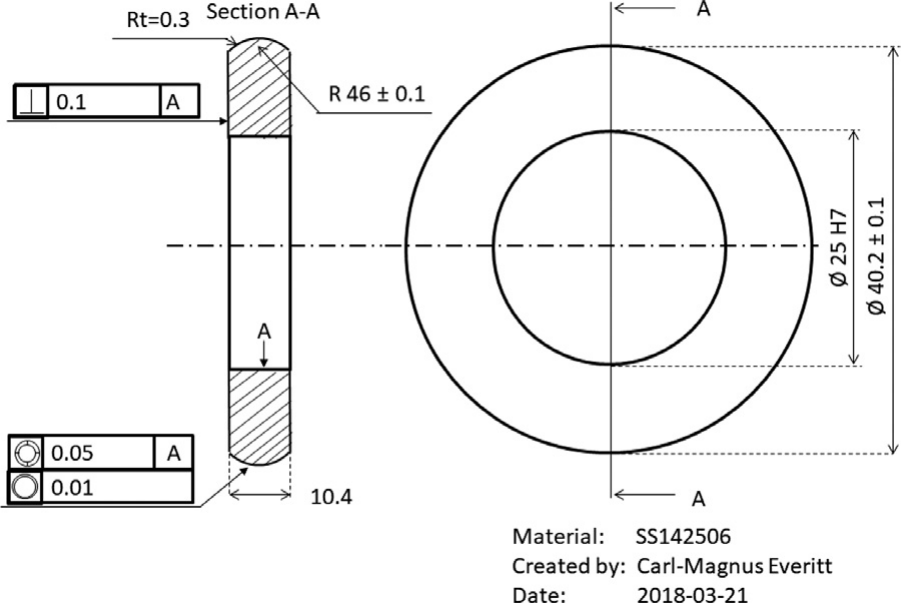
The counter-disc.

**Fig. B1:**
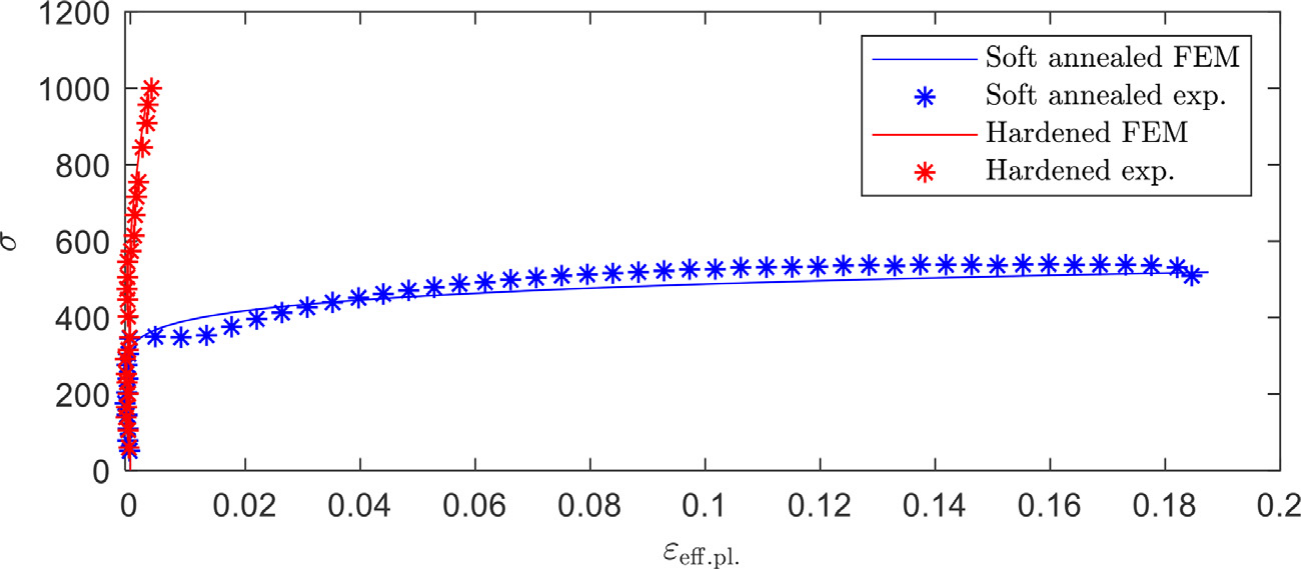
Tensile curves for SS142506.

**Table B1:** Tensile material parameters for SS142506.

Description	Symbol/unit	Soft annealed	Hardened
Young's Modulus	*E/*GPa	210	210
Poisons ratio	*ν/*-	0.3	0.3
Initial yield stress	*σ*_y0/_MPa	320	600
Hardening exponent	*n/*-	0.1	0.5
Reference strain	*ε_0/_*10^−3^	1.5	2.9

**Fig. C1:**
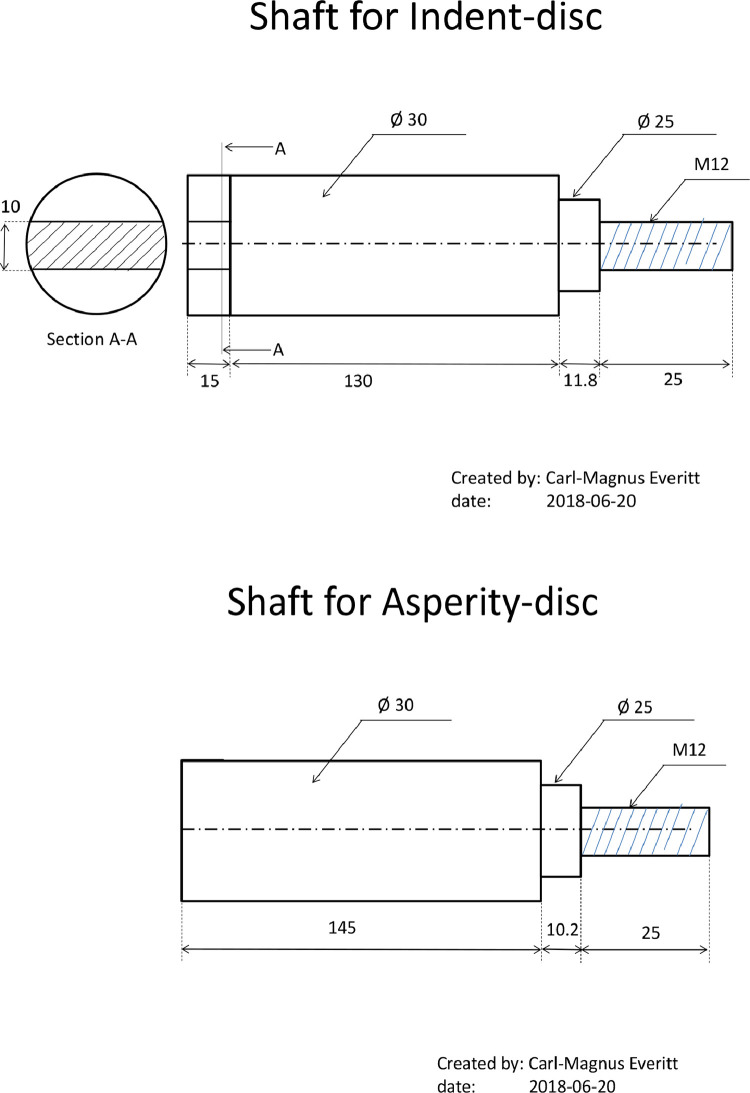
Drawings of the axis used in the set-up for creating the discs. The shaft for the indent-disc contains a tangible section to the left.

## Appendix A Drawings of the discs

Figs. A1, A2, A3 present drawings of the tree different discs used in the experimental method. Different designs were needed for the different disc types. The indent-disc should be slightly wider than the asperity-disc in order to avoid edge effects. The counter-disc needed to be crowned in order to allow for small alignment errors in the twin-disc test rig and to attain the required test contact pressure. The diameter of the asperity- and counter-discs as well as the crowning of the counter-disc were determined for the maximum Hertz pressure p_H_ = 2.3 GPa in the twin-disc test rig.

## Appendix B Material for asperity and counter-disc

The material data was obtained from the literature [28]. Fig B1 shows the tensile curves for the material in the soft annealed state as delivered from suppliers and in the hardened conditions. The curves were fitted to the Swift material model

(B.1) $$\sigma_{y}=\sigma_{y0}{\left(1+\frac{\varepsilon_{\text{eff}.\text{pl}}}{\varepsilon_{0}}\right)}^{n}$$

where *σ*_y0_ is the initial yield stress, *n* is the hardening exponent, *ε_0_* the reference strain and *ε*_eff.pl_ the effective plastic strain. The material parameters are presented in Table B1. The compressive behaviour of the material was for simplicity assumed to be equal to the tensile behaviour.

## Appendix C Drawings of the shafts

Drawings of the shafts used during the making of the discs are presented in Fig. C1. To make it easier to rotate the shaft for the indent-disc, it had a tangible section on the left side.
